# Quality of life in patients with cervical cancer between the Han nationality and ethnic minorities in the Yunnan Province of China

**DOI:** 10.1186/s12905-023-02240-3

**Published:** 2023-05-03

**Authors:** Min Zhao, Xin Pu, Guo-Yu Ma, Meng-Jiao Zhang, Lei Luo, Rong-yan Gu, Ming-Zhu Gao, Le Cai

**Affiliations:** 1 School of Public Health, Kunming Medical University, 1168 Yu Hua Street Chun Rong Road, Cheng Gong New City, Kunming, 650500 China; 2 Medical Administration Department, The Third Affiliated Hospital of Kunming Medical University (Yunnan Cancer Hospital, Yunnan Cancer Center), Xi Shan County, 519 Kun Zhou Road, Kunming, 650118 Yunnan China; 3 Medical Records Statistics Department, The First Affiliated Hospital of Kunming Medical University, 295 Xi Chang Road, Kunming, 650032 Yunnan China; 4 Yunnan Cancer Center Office, The Third Affiliated Hospital of Kunming Medical University (Yunnan Cancer Hospital, Yunnan Cancer Center), Xi Shan County, 519 Kun Zhou Road, Kunming, 650118 Yunnan China

**Keywords:** Cervical cancer, Health-related quality of life, FACT-Cx, Ethnic minorities, China

## Abstract

**Background:**

Cervical cancer is the fourth most diagnosed cancer and the leading cause of cancer death, and it still poses a crippling threat to women’s health. China launched the National Cervical Cancer Screening Program for Rural Women in 2009, and an increasing number of cervical cancer patients have been detected. Health-related quality of life is not only the end point of cancer research but is also related to socioeconomic and clinical factors and has received an increasing amount of attention. In light of the characteristics of the Yunnan nationality, we conducted cross-sectional research to assess and explore the health-related quality of life in both Han and ethnic minority patients.

**Methods:**

A cross-sectional study was conducted from January 2020 to May 2021 at the Third Affiliated Hospital of Kunming University/Yunnan Cancer Hospital. Patients, including 100 Han patients and 100 ethnic minorities, were interviewed using the FACT-Cx questionnaire within 3 months of receiving treatment.

**Results:**

Patients of Han ethnicity and ethnic minorities were comparable in both sociodemographic and clinical features. The total FACT-Cx scores were 139.38 ± 9.83 and 134.39 ± 13.63 in Han and ethnic minority patients, respectively (*P* < 0.05). Significant differences were shown in physical well-being, emotional well-being and the FACT-Cx subscale between the Han and ethnic minority groups. Independent predictors of the FACT-Cx scale were ethnicity, educational level, participation in the National Cervical Cancer Screening Program for Rural Areas (NCCSPRA) and clinical stage.

**Conclusions:**

The results of our study imply that the HRQOL of Han patients is better than that of ethnic minority patients. Thus, clinicians and related health workers should pay more attention to the HRQOL of cervical cancer patients, especially for ethnic minority patients, and provide psychosocial interventions as much as possible to improve their HRQOL. Policies should also aim to strengthen health education regarding cervical cancer and expand the coverage of the NCCSPRA among those who are ethnic minorities, are older and have low educational levels.

## Introduction

Cervical cancer is the fourth most frequently diagnosed cancer and the fourth leading cause of cancer death in women, with 604,000 new cases and 342,000 deaths worldwide in 2020 [1]. Cervical cancer represents a major threat to women’s health [2]. The proportion of women with cervical cancer who die from the disease is greater than 60% in many low- and middle-income countries, which is more than twice the proportion in many high-income countries, where it is as low as 30% [3]. The incidence and mortality rates in transitioned countries are 11.3/100,000 and 5.2/100,000 thousand, respectively [1].

Cervical cancer ranks as the second most common female malignant tumor in China, accounting for 18.6% of all cancer patients [4]. Data showed that China’s crude incidence rate (CIR) and crude mortality rate (CMR) of cervical cancer were 16.56/100,000 and 5.04/100,000 in 2015 [5]. The incidence and mortality of cervical cancer in China are still at slightly high levels worldwide [5]. Disparities in incidence and mortality are not only observed between developing and developed countries but also found in different areas within a country [1, 6]. Cervical cancer is a serious issue in women’s health and has been a heavy burden to China [5]. From 2011 to 2015, the CIR and CMR of cervical cancer in Yunnan Province were 17.39/100,000 and 5.14/100,000, respectively; both rates were higher than those in China and some Western countries [6]. Up until 2016, the CIR and CMR of cervical cancer in Yunnan Province were 16.01/100,000 and 5.39/100,000, respectively [7].

However, cervical cancer is considered nearly completely preventable because of the highly effective primary (HPV vaccine) and secondary (screening) prevention measures [1]. HPV vaccines are currently available in China, but they remain difficult to obtain, especially for women in ethnic minority areas. In addition, the price of the HPV vaccine is not cheap, ranging from 1815 to 3969 RMB (275 to 601 USD). High-quality screening programs are also important to prevent cervical cancer among unvaccinated women and for oncogenic subtypes not covered by the vaccine [1]. Although screening with cytology and high-risk human papillomavirus DNA testing have reduced the incidence and mortality of cervical cancer, women who do not have access to health care and those living in resource-poor areas remain at high risk of death from cervical cancer [8].

In 2009, China launched the National Cervical Cancer Screening Program for Rural Areas (NCCSPRA), providing free screening for rural women aged 15 to 64 years; its purpose is to encourage early diagnosis, detection, prevention, and treatment. Nevertheless, the uptake of cervical cancer screening is associated with local economic status, household income, education, employment, health insurance, and health check-up [9]. The screening rate of cervical cancers in Yunnan Province is lower than China’s average level [10]. Earlier detection and more effective treatment regimens have resulted in a tremendous increase in cervical cancer survivors over the last decades [11]. This means that cervical cancer patients can be diagnosed at early stages; therefore, cancer survivors have also increased correspondingly and lived longer than before [12]. It is important to simultaneously assess quality of life while measuring progression-free and overall survival [8].

Health-related quality of life (HRQOL) is defined by the World Health Organization (WHO) as “the perception of the individual about his position in life, in the context of culture and value systems in which he lives, and about his goals, expectation, standards, and concerns” [13]. HRQOL is an important end point for clinical oncology research [14], and it is also regarded as one of the key outcome indicators in oncology [15]. HRQOL is a complex, multidimensional construct with a range of conceptual definitions [16]. HRQOL is derived from the general concept of quality of life and has been developed specifically to indicate quality of life as it relates to diseases or treatment. HRQOL is affected not only by disease and treatment but also by personal factors and social networks [17]. In recent years, the HRQOL of patients with cervical cancer and precancerous lesions has attracted attention domestically and overseas [18–20]. The HRQOL of cervical cancer patients is associated with many factors, such as age, education level, occupation, level of annual household income, marital status, number of pregnancies and therapy methods [21–24]. Relevant studies also show that the HRQOL score of cervical cancer patients in Yunnan Province is low, especially for female patients after radiotherapy and chemotherapy [25–27]. However, the HRQOL of ethnic minority patients with cervical cancer has not received enough attention to date in Yunnan Province. Moreover, the HRQOL of patients with cervical cancer among different minorities is still poorly understood in China, especially in Southwest China.

Yunnan Province is the province with the largest number of ethnic groups and is located in Southwest China. There are 15.63 million ethnic minorities in Yunnan, accounting for 33.12% of the total population [28]; most of these individuals are residents in areas that border other countries. In a report on China’s provincial economic comprehensive competitive development (2015–2016), China’s 31 provinces were categorized into upstream, midstream, and downstream regions; according to the report, Yunnan province is in the downstream group [29].

In China, the coverage of free cervical cancer screening is limited, and lesbian or bisexual individuals often mistakenly believe that cervical cancer screening is unnecessary [30]. For various reasons, cervical cancer can be found at an advanced stage. The treatment of cervical cancer will change women's physiological characteristics and affect their perception of body image and sexual function. Chemotherapy causes various adverse reactions, such as nausea, vomiting, diarrhea, alopecia, sallowness, emaciation, hormone changes, and other negative emotions, such as depression, anxiety, and suicidal thoughts and actions, which may lead to a reduction in the quality of life of cervical cancer patients. Due to changes in the medical model, medicine has changed from traditional disease diagnosis and treatment to the prevention, recuperation, rehabilitation and improvement of patients' quality of life as the ultimate goal. Since it is possible to prolong the life of patients with cervical cancer, it is certain to become an inevitable trend to focus on the quality of life of patients with cervical cancer. Therefore, the aim of our study was to assess HRQOL among patients with cervical cancer in Yunnan Province, specifically, to explore the influencing factors of HRQOL between Han and ethnic minority patients and to provide a reference for improving HRQOL among patients.

## Methods

### Study area and participants

Our study was conducted from January 2020 to May 2021 in the Third Affiliated Hospital of Kunming Medical University/Yunnan Cancer Hospital, which is a tertiary tumor-specialized hospital. The hospital mainly provides medical services to patients in Yunnan Province. Selection of samples: From January 2020 to May 2021, 106 ethnic minority patients were admitted to the Third Affiliated Hospital of Kunming Medical University/Yunnan Cancer Hospital.However, 6 of them failed to complete the whole investigation or the data were incomplete, so there were 100 cases. At the same time, 100 Han patients with the same basic conditions were selected for investigation. Therefore, this study including 100 Han patients and 100 ethnic minorities, who were interviewed using the FACT-Cx questionnaire within 3 months of receiving treatment.

The inclusion criteria were patients who were (a) aged 18 years or above, (b) diagnosed pathologically with cervical cancer or precancerous lesions, (c) previously untreated and newly diagnosed, and (d) identified with a definite clinical stage.

The exclusion criteria were patients who (a) had severe comorbidities, (b) had other malignant tumors, (c) had contraindications before radiotherapy, chemotherapy, and surgery, (d) had cognitive dysfunction and (e) refused to participate in the interview.

### Questionnaire

The Functional Assessment of Cancer Therapy-cervix cancer (FACT-Cx) (Chinese version 4.0) was applied to assess QOL in our study. FACT-Cx is derived from the FACT-G (general module of the Functional Assessment of Cancer Therapy) and is especially used to assess the QOL of cervical cancer patients. The FACT-G is composed of 27 items and covers four dimensions: physical well-being (PWB, 7 items), social/family well-being (SWB, 7 items), emotional well-being (EWB, 6 items) and functional well-being (FWB, 7 items). The FACT-Cx contains 15 additional items specifically related to cervical cancer, such as symptoms, sexuality, treatment sequelae, and psychological activities. Each item is scored on a five-point scale (0 = not at all; 1 = a little bit; 2 = somewhat; 3 = quite a bit; 4 = very much) [8]. The total score of the questionnaire may vary between 0 and 168 [31]. The higher the score is, the better the QOL of the patient is. The FACT-Cx has good reliability, validity, responsiveness and feasibility and can be used to evaluate the QOL of patients in China [15, 32].

The questionnaire also included sociodemographic and clinical characteristics. Demographic characteristics included age, education level, occupation, having a spouse or not, number of pregnancies, monthly household income, status of awareness and participation in the NCCSPRA. Clinical characteristics included clinical stages and pathological stages.

### Data collection

Interviewers were graduate students at Kunming Medical University and doctors at Yunnan Cancer Hospital; they were trained so that they could understand the research background and questionnaire contents well. The purpose was explained to the patients before face-to-face interviews were held, and informed consent was obtained from all patients. In addition, clinical information was collected from the patients’ electronic medical records.

### Ethical approval

This study was approved by the Ethics Committee of Yunnan Cancer Center (Yunnan Cancer Hospital & The Third Affiliated Hospital of Kunming Medical University). Informed consent was signed by eligible patients before the investigation.

### Statistical analysis

Epidata3.1 software was used to establish the database, and questionnaires were inputted by the double entry method. SPSS26.0 (Statistical Product and Service Solutions 26.0) software was applied for data analysis.

Measurement data are expressed as the mean and standard deviation. When necessary, measurement data are also reported as enumeration data, such as for age. Enumeration data were described by frequencies and treated with the chi-square test. Measurement data were analyzed with t tests and ANOVA, and pairwise comparisons were conducted for further comparisons between groups.

The statistical significance level was set at 0.05, and all statistical tests were 2-tailed.

We compared the HRQOL of patients with cervical cancer and precancerous lesions between Han and ethnic minority people. We also compared the HRQOL of different subgroups divided by sociodemographic and clinical characteristics within Han and ethnic minority patients. Multiple linear regression analysis was used to identify predictors of HRQOL.

## Results

A total of 200 patients were interviewed, namely, 100 Han and 100 ethnic minority patients. The age of the patients ranged from 26 to 68 years old, with an average age of 45.57 ± 8.99 years.

Table 1 presents the comparison of sociodemographic and clinical characteristics between the Han and ethnic minority groups. The results showed that Han and ethnic minority patients were comparable.

**Table 1:** Comparison of sociodemographic and clinic characters between Han and ethnic minority groups

Variables	Ethnicity		Total	${\chi }^{2}$	*P* value
	Han N (%)	Ethnic Minority N (%)			
Age groups (years)					
20–39	23(23.0)	26(26.0)	49(24.5)	0.385	0.825
40–49	45(45.0)	41(41.0)	86(43.0)		
≥ 50	32(32.0)	33(33.0)	65(32.5)		
Education levels					
Illiterate (0)	16(16.0)	23(23.0)	39(19.5)	1.601	0.659
(Years of education)					
Primary school (1–6)	30(30.0)	28(28.0)	58(29.0)		
Junior middle school (7–9)	31(31.0)	29(29.0)	60(30.0)		
High school or higher (≥ 10)	23(23.0)	20(20.0)	43(21.5)		
Occupation					
Farmer	63(63.0)	64(64.0)	127(63.5)	0.022	0.883
Others	37(37.0)	36(36.0)	73(36.5)		
Have a spouse					
Yes	93(93.0)	92(92.0)	185(92.5)	0.072	0.788
No	7(7.0)	8(8.0)	15(7.5)		
Number of pregnancies (times)					
≤ 3	56(56.0)	61(61.0)	117(58.5)	0.515	0.473
> 4	44(44.0)	39(39.0)	83(41.5)		
Monthly household income (RMB)					
≤ 2000	32(32.0)	33(33.0)	65(32.5)	0.213	0.975
2001–3000	23(23.0)	21(21.0)	44(22.0)		
3001–7000	22(22.0)	21(21.0)	43(21.5)		
≥ 7001	23(23.0)	25(25.0)	48(24.0)		
Know the NCCSPRA					
Yes	42(42.0)	35(35.0)	77(38.5)	1.035	0.309
No	58(58.0)	65(65.0)	123(61.5)		
Participation in the NCCSPRA					
Yes	29(29.0)	21(21.0)	50(25.0)	1.707	0.191
No	71(71.0)	79(79.0)	150(75.0)		
Know the HPV vaccine					
Yes	38(38.0)	33(33.0)	71(35.5)	0.546	0.460
No	62(62.0)	67(67.0)	129(64.5)		
Mode of case-detecting					
Clinical symptoms	61(61.0)	58(58.6)	119(59.8)	0.931	0.628
Physical examination	17(17.0)	22(22.2)	39(19.6)		
Screening	22(22.0)	19(19.0)	41(20.6)		
Clinical stages					
CIN	37(37.0)	38(38.0)	75(37.5)	0.115	0.990
Stage I	30(30.0)	28(28.0)	58(29.0)		
Stage II	18(18.0)	18(18.0)	36(18.0)		
Stage III and above	15(15.0)	16(16.0)	31(15.5)		
Pathological classification					
Squamous cell carcinoma	46(47.9)	47(47.0)	93(47.4)	0.038	0.981
Adenocarcinoma	10(10.4)	10(10.0)	20(10.2)		
Other Types	40(41.7)	43(43.0)	8(42.3)		
Insurance type					
Employee medical insurance	14(14.0)	20(20.0)	34(34.0)	1.472	0.479
Residents’ medical insurance	63(63.0)	61(61.0)	124(124.0)		
Other insurances	23(23.0)	19(19.0)	42(42.0)		
Treatment					
LEEP/CKC	28(28.0)	34(34.0)	62(31.0)	4.981	0.173
Radical hysterectomy	45(45.0)	30(30.0)	75(75.0)		
Concurrent chemoradiotherapy	20(20.0)	28(28.0)	48(48.0)		
Postoperative adjuvant therapy	7(7.0)	8(8.0)	15(15.0)		
Total	100(50.0)	100(50.0)	200(100)		

Approximately one-fifth of patients were illiterate, 63.5% were farmers, 92.5% had a spouse, and 58.5% had fewer than 3 pregnancies. In addition, 54.5% had a monthly income of less than 3000 RMB (approximately 465 US).

At the time of interview, 38.5% were aware of the NCCSPRA, 25.0% participated in the screening, and 35.5% were aware of the HPV vaccine. Approximately 60% of patients’ cancer was detected through abnormal clinical symptoms. In terms of clinical characteristics, 37.5% were CIN, 29% were stage I, 18% were stage II, and 15.5% were stage III or above; 47.4% of them were diagnosed with squamous cell carcinoma, 10.2% were adenocarcinoma, and 42.3% were other types. The proportions of insurance types and treatments were also similar in the two groups. The results indicated that patients of Han ethnicity and ethnic minorities were comparable in regard to both sociodemographic and clinical features.

Table 2 presents the comparison results of the FACT-Cx scale between Han and ethnic minorities.

**Table 2:** Comparison results of the FACT-Cx scale between Han and ethnic minorities (X ± S)

FACT-CX scale	Han	Ethnic Minority	Total	t	*P* value
PWB	24.38 ± 3.39	22.88 ± 4.67 *	23.63 ± 4.14	2.600	0.010
SWB	26.99 ± 2.16	26.45 ± 2.83	28.00 ± 2.53	1.515	0.131
EWB	21.11 ± 2.64	19.94 ± 3.22 **	20.53 ± 2.99	2.812	0.005
FWB	18.96 ± 4.62	18.65 ± 4.92	18.81 ± 4.76	0.460	0.646
CxS	47.94 ± 3.49	46.47 ± 4.59 *	47.21 ± 4.13	2.549	0.012
Total score of FACT-Cx	139.38 ± 9.83	134.39 ± 13.63 **	136.89 ± 12.11	2.970	0.003

^**^: *P* < 0.01, *: *P* < 0.05, indicating that there are statistical significances between different subgroups of HROL of Han nationality and ethnic minorities

The total FACT-Cx scores were 139.38 ± 9.83 and 134.39 ± 13.63 for Han and ethnic minority patients, respectively (*P* < 0.05), indicating that the HRQOL of Han patients was better than that of ethnic minority patients. In the comparison of subdimensions, significant differences were shown in PWB (24.38 ± 3.39 vs. 22.88 ± 4.67), EWB (21.11 ± 2.64 vs. 19.94 ± 3.22) and Cxs (47.94 ± 3.49 vs. 46.47 ± 4.59) between the Han and ethnic groups, whereas the SWB (26.99 ± 2.16 vs. 26.45 ± 2.83, *P* = 0.131) and FWB (18.96 ± 4.62 vs. 18.65 ± 4.92, *P* = 0.646) scores did not differ significantly between the two groups.

We categorized the interviewees into different subgroups by sociodemographic and clinical characteristics and explored the differences in their HRQOL. Table 3 presents a comparison of HRQOL in different subgroups of Han and ethnic minority patients.

**Table 3:** Comparison of HRQOL in different subgroups in Han and ethnic minority groups

Variables	Han			Ethnic Minority		
		*t/F*	*P* value		*t/F*	*P* value
Age groups (years)						
20–39	138.43 ± 12.34	0.457	0.635	141.35 ± 11.10	5.583	0.005
40–49	140.42 ± 7.66			133.41 ± 13.41		
≥ 50	138.59 ± 10.69			130.12 ± 13.94 **		
Education level						
Illiterate	138.69 ± 8.99	2.64	0.054	130.00 ± 13.22	6.648	< 0.001
Primary school	137.43 ± 10.20			128.07 ± 14.50		
Junior middle school	138.00 ± 9.37			139.45 ± 10.81		
High school or higher	144.26 ± 9.45			140.95 ± 11.32 **		
Occupation						
Farmer	137.60 ± 10.38	-2.417	0.018	133.11 ± 13.66	-1.256	0.212
Others	142.41 ± 8.06 *			136.67 ± 13.47		
Have a spouse						
Yes	140.02 ± 9.07	-2.439	0.017	134.28 ± 13.53	0.266	0.791
No	130.86 ± 15.52 *			135.63 ± 15.68		
Number of pregnancies (times)						
≤ 3	139.61 ± 10.06	0.853	0.796	135.97 ± 14.23	1.455	0.149
> 4	139.09 ± 9.63			131.92 ± 12.42		
Monthly household income (yuan)						
≤ 2000	138.66 ± 8.07	2.201	0.093	133.30 ± 13.38	0.935	0.427
2001–3000	140.04 ± 11.03			131.05 ± 13.85		
3001–7000	135.86 ± 11.54			136.38 ± 12.05		
≥ 7001	143.09 ± 8.13			136.96 ± 14.99		
Know the NCCSPRA						
Yes	142.83 ± 7.57	3.12	0.002	138.74 ± 11.42	2.399	0.018
No	136.88 ± 10.55 **			132.05 ± 14.22 *		
Participation in the NCCSPRA						
Yes	142.31 ± 7.06	2.276	0.026	140.57 ± 11.00	2.735	0.009
No	138.18 ± 10.56 **			132.75 ± 13.85 **		
Know the HPV vaccine						
Yes	140.63 ± 11.62	0.997	0.321	141.00 ± 9.82	3.604	< 0.001
No	138.61 ± 8.56			131.13 ± 14.12 **		
Model of case-detecting						
Clinical symptoms appear	137.75 ± 10.80	2.312	0.105	131.72 ± 14.28	2.764	0.068
Physical examination	142.76 ± 8.10			136.55 ± 11.15		
Screening	141.27 ± 7.15			139.42 ± 12.97		
Insurance type						
Employee medical insurance	144.79 ± 9.13	3.628	0.030	142.10 ± *9.43*	4.293	0.016
Residents’ medical insurance	137.60 ± 10.38			132.66 ± 13.67		
Others	140.96 ± 7.16 *			131.84 ± 14.90 *		
Clinical stages						
CIN	141.32 ± 7.17	4.114	0.009	141.47 ± 9.73	7.677	< 0.001
Phase I	142.10 ± 8.61			129.07 ± 14.47		
Phase II	134.17 ± 12.17			134.22 ± 11.32		
Phase III and above	135.40 ± 11.79 **			127.06 ± 15.27 **		
Pathological classification						
Squamous cell carcinoma	137.00 ± 11.99	2.441	0.093	128.30 ± 14.16	10.872	< 0.001
Adenocarcinoma	141.1 ± 7.59			137.30 ± 11.20		
Other Types	141.50 ± 7.07			140.37 ± 10.55 **		
Treatment						
LEEP/CKC	141.11 ± 7.79	4.254	0.007	141.15 ± 10.12	6.224	0.001
Radical hysterectomy	140.89 ± 7.65			134.27 ± 13.81		
Concurrent chemoradiotherapy	132.70 ± 14.44			128.54 ± 13.64		
Postoperative adjuvant therapy	139.38 ± 9.83 **			126.63 ± 14.49 **		

^**^: *p* < 0.01, ^*^: *p* < 0.05, indicating that there are statistical significances between different subgroups of HRQOL of Han nationality and ethnic minorities^*^

Significant differences in HRQOL were found in different age groups and educational levels of ethnic minority patients (*P* < 0.01). Poor HRQOL was found in ethnic patients with older age and a low education level. Significant differences in HRQOL were found in different occupations (farmer vs. others) and marriage statuses in Han patients (*P* < 0.05). In Han patients, low HRQOL was found in farmers and patients without spouses.

In our study, we also investigated the awareness of or participation in the NCCSPRA and HPV vaccines. We found that the awareness and participation of the NCCSPRA had a positive effect on HRQOL. Both Han and ethnic minority patients who knew and participated in the NCCSPRA reported better HRQOL than those who did not (*P* < 0.05). In addition, ethnic minority patients with knowledge of the HPV vaccine reported better HRQOL than those who did not know about it (*P* < 0.01).

Our study is also concerned about the influence of different insurance types. The results demonstrated that patients with employee insurance had the best HRQOL compared with other insurance types in Han and ethnic minority patients (*P* < 0.05). A significant difference was found between employee insurance and residents’ medical insurance in further pairwise comparisons of both Han and ethnic minority patients (*P* < 0.05); the HRQOL scores between employee insurance and other types of insurance were also found to be significantly different in ethnic minority people (*P* < 0.05). Pairwise comparison results are not shown in the table.

Poor HRQOL was found in patients with advanced-stage disease in both Han and ethnic minority groups compared with patients with early-stage disease (*P* < 0.01). Moreover, patients with CIN in both groups had the highest HRQOL scores (141.32 ± 7.17 and 141.47 ± 9.73 in the Han and ethnic minority groups, respectively). In addition, the HRQOL of patients with squamous cell carcinoma, adenocarcinoma, and other pathological classifications were 128.30 ± 14.16, 137.30 ± 11.20 and 140.37 ± 10.55, respectively, in the ethnic minority group, with a significant difference (*P* < 0.001). Patients who experienced LEEP/CKC had the best HRQOL scores (141.11 ± 7.79 and 141.15 ± 10.12 in Han and ethnic minority patients, respectively).

The results of multiple linear regression analysis for all groups are shown in Table 4. Independent predictors of the FACT-Cx scale were ethnicity, educational level, participation in NCCSPRA and clinical stage.

**Table 4:** Multiple linear regression analysis of HRQOL in patients with cervical cancer and precancerous lesion

Variables	Regression coefficient *β*	Standard error *S.E*	Standardization coefficient *β*	*t*	*P* value	R^2^	*F*	*P* value
Ethnicity (reference: Han)	-4.202	1.583	-0.173	-2.654	0.009	0.189	12.337	< 0.001
Education level (reference: Illiterate)	2.649	0.790	0.225	3.352	0.001			
Primary school	-2.474	2.262	-0.093	-1.094	0.275			
Junior middle school	3.471	2.244	0.132	1.547	0.123			
High school or higher	5.907	2.495	0.201	2.367	0.008			
Participated in screening (Reference: Yes)	-4.632	1.847	-0.165	-2.507	0.013			
Clinic stages (Reference: CIN)	-2.414	0.766	-0.214	-3.150	0.002			

## Discussion

Assessment of quality of life is important for evaluating the full impact of cancer therapies on the well-being of patients [33]. Considering the growing numbers of cervical survivors, understanding the specific factors that influence HRQOL for this population is necessary [34]. It has been previously suggested that influential factors of HRQOL of cervical cancer patients include stage, treatment, social support, response to the disease, and personal factors related to age, educational level, economic status, and bad living habits [35].

The overall HRQOL in our study was better than that found in research conducted both in China and other countries [8, 15, 17, 20, 21, 23, 31, 33, 36–40]. We noticed that most patients were at an advanced stage in some clinical drug trials in other countries [8, 21, 33, 39, 40] and without CIN. Patients diagnosed at an early stage showed better HRQOL when receiving neither chemotherapy nor radiation therapy [37]. The proportions of CIN and stage I were 37.5% and 29.0%, respectively, in our research. Poor HRQOL was found in patients with advanced-stage cancer, with the lowest scores for Phase III and above HRQOL-(135.40 ± 11.79) for Han nationality and (127.06 ± 15.27) for ethnic minorities-and one of the independent predictors of HRQOL was clinical stage. Therefore, a high FACT-Cx score may have been related to the clinical stages of the patients in our study.

The total HRQOL score was lower than that found in another similar study conducted in Shanghai [41] for the following reasons. First, the research site was Shanghai, where the society and economy are well developed; second, there were only 2 patients with stage III disease and were no patients with stage IV disease in the research conducted in Shanghai.

The results of our study indicated that Han patients have a better quality of life than ethnic minority patients, which is inconsistent with a similar study conducted in Yunnan [38]. However, we also noticed that the sample size of ethnic minority patients was only 21 in that study, which may have contributed to the different conclusions.

It is believed that cultural values or beliefs held by ethnic minorities have impacts on individuals’ thoughts, interpretations and behavior [42]. Sang Shuping’s research on the general population in Yunnan demonstrated that there is an illusion about health in ethnic minorities; most of them think that health is only the absence of physical disease, and thus pay less attention to mental health, such as pressure and anxiety levels [43]. There is also a popular belief in mainland China that cervical cancer is associated with undesirable sexual habits and hygiene [17]. Ethnic minority patients often feel much discomfort in the process of treatment because of their lack of medical concepts, sense of urgency and shame about gynecological diseases. Furthermore, the decline of sexual attraction, the onset of an inferiority complex, etc., may affect the normal social interactions of patients. All of these false beliefs about health and disease are especially common in ethnic groups and help to deteriorate their HRQOL. Interventions should be advocated to establish the right beliefs, to impart correct information about the disease, to conduct psychosocial interventions, and even to help patients return to normal functional well-being.

Other research conducted in a neighboring province (Guizhou) also revealed that ethnic minority areas are economically backward and have poor hygiene [44], which is similar to the situation in Yunnan. Early marriage and childbirth are common among ethnic minorities. It is common for women to be more involved in family affairs and housework in China, especially for ethnic minority women in Yunnan who engage in agricultural production. Therefore, cervical cancer can impose a crippling effect on their role in the family. The cultural values or beliefs held by ethnic minorities may lead to low overall HRQOL.

Ethnic minority patients reported lower scores in physical and emotional well-being on an additional subscale for cervical cancer (*P* < 0.05, Table 2). The results suggest that ethnic minority patients are more vulnerable in those domains when faced with cervical cancer. Both physical symptoms and emotional distress can affect a patient’s quality of life [45]. Physical impairments in Chinese cervical cancer survivors include poor sleep quality, a loss of appetite, vomiting, and a loss of fertility [42]. In Chinese culture, the family is expected to be the main source of one’s emotional and practical support; the more support the patient receives from family members, the higher the HRQOL achieved by the patient [17]. However, for ethnic minority people, it is common for males to migrate from their home to another place in the workforce in the process of urbanization; thus, spouses may not live with their wives and thus may not support patients emotionally well. Therefore, ethnic patients may receive less emotional support from their spouses. Ethnic minorities also reported lower CxS scores than Han patients. The FACT-Cx additional subscale can assess the late effects of cervical cancer and its treatment, which is also a significant predictor influencing survival for patients [37].

It is worth noting that EWB and FWB scored the lowest among the five subdimensions in our research. This outcome is consistent with then findings of similar studies [15, 17, 23, 24, 38, 46]. Functional well-being measures activity level, sleep, and overall life satisfaction, while emotional well-being assesses depression, anxiety, and other affective responses to illness and treatment [47]. Ding’s research in China also suggested that we should pay more attention to patients’ FWB [17]. One explanation for this may be due to Chinese women’s role in society and family. Most Chinese patients are more likely to return to work or continue to farm after illness. Cervical cancer can badly damage their functional well-being.

In the comparison of different subgroups in ethnic minority patients, it was found that as age increased, HRQOL decreased; furthermore, HRQOL improved as education level increased. Patients with higher education levels are more likely to communicate with clinical workers and their families, which is conducive to the elimination of depression and negative emotions [48]. As a result, they have a higher quality of life.

Nevertheless, Han patients reported lower scores than patients with other occupations because farming in China is often seen as a low-paid and laborious occupation; Han patients with a spouse scored higher than those without a spouse.

Our study highlighted that the awareness of and participation in the NCCSPRA had a positive impact on HRQOL in both Han and ethnic minority patients. Cervical cancer screening is recognized as an effective intervention to prevent the occurrence of advanced disease and death from cervical cancer [2]. However, data have shown that the cervical cancer screening rate in Yunnan is lower than China’s average level; in addition, the screening rate in ethnic groups is lower than that in Han people [10]. It is presumed that patients with an awareness of those who participate in the NCCSPRA are more likely to be concerned about their health and more likely to gain related disease information. That is why those patients had higher HRQOL scores. Therefore, expanding the coverage of NCCSPRA is necessary, especially for areas with poor economic development and limited health resources. Screening programs should give priority to those who are ethnic minorities, are older, and have low educational levels. Only in this way can patients be found at an early stage. Women with low education levels could be given a greater chance of accessing the screening program.

Moreover, ethnic patients who knew about the HPV vaccine had better HRQOL than those who did not, which was an important finding in our study. It is likely that patients who knew about the HPV vaccine also had a strong sense of health; thus, they might pay more attention to cervical cancer-related knowledge and, in turn, have better HRQOL. There is currently a large gap between the supply of and demand for HPV vaccines in China; thus, health bureaus should pay attention and make efforts to address this problem.

Our research also divided patients according to their insurance types. China's medical insurance is mainly composed of medical insurance for employees and for residents, with different reimbursement rates. Patients with employee medical insurance often have higher reimbursement rates than those with residents’ insurance. Therefore, patients with employee medical insurance reported better HRQOL scores than those with other insurance types.

In general, both Han and ethnic minority patients showed the same trend in HRQOL in regard to clinical stages and treatment. Treatment is strongly associated with clinical stage. Patients with early-stage disease showed significantly better HRQOL than advanced-stage patients. However, the difference in HRQOL was not observed in different models of case detection in our research. Health education of cervical cancer should be conducted to improve women's lifestyle and beliefs, which is helpful for preventing and controlling cervical cancer [47, 49]. Health education for cervical cancer should be strengthened, especially for ethnic women with low education levels and older age.

## Conclusions

There are few studies about the HRQOL of cervical cancer between Han and minority patients in Yunnan. The results of our study demonstrate that differences in the HRQOL of cervical cancer do exist between Han and ethnic minority patients. Clinicians and related health workers should pay more attention to the HRQOL of cervical cancer patients, especially for ethnic minority patients, and provide psychosocial interventions as much as possible to improve their HRQOL. Related policies should also aim to strengthen the health education on cervical cancer and expand the coverage of the NCCSPRA among those who are ethnic minorities, are older and have low educational levels. Related departments should make greater efforts to increase the HPV vaccine supply.

### Limitations

There were still some limitations in our study. First, the use of the cross-sectional survey approach limits the inference of causality and the extension of the research results. Further research is needed to explore the relationship between the variables. Second, our research still lacks an objective evaluation index of the HRQOL of patients, which may affect the accuracy of the HRQOL. Third, the inclusion of ethnic minorities is imprecise because there are 55 ethnic minorities other than the Han nationality present in China. Fourth, comorbidity and quality of sexual life are not included in our research, which might be another important factor influencing HRQOL. Fifth, the use of a blank control is not included in this study.

## Data Availability

The scale used in this study has been found to be reliable and applicable after reliability and validity tests. The datasets generated and/or analyzed during the current study are not publicly available because they are related to patients; however, they are available from the corresponding author upon request.

## Abbreviations

HRQOL: Health-related quality of life
CIR: Crude incidence rate
CMR: Crude mortality rate
HPV: Human papilloma virus
NCCSPRA: The National Cervical Cancer Screening Program for Rural Women
CIN: Cervical intraepithelial neoplasia
LEEP: Loop electrosurgical excision procedure
CKC: Cold knife conization
ANOVA: One-way analysis of variance
PWB: Physical well-being
SWB: Social/family well-being
EWB: Emotional well-being
FWB: Functional well-being
CxS: Cervical cancer subscale
